# Valorization of whey proteins and beetroot peels to develop a functional beverage high in proteins and antioxidants

**DOI:** 10.3389/fnut.2022.984891

**Published:** 2022-12-14

**Authors:** Eman M. Abdo, Marwa G. Allam, Mohamed A. E. Gomaa, Omayma E. Shaltout, Hanem M. M. Mansour

**Affiliations:** ^1^Department of Food Science, Faculty of Agriculture, Saba Basha, Alexandria University, Alexandria, Egypt; ^2^Department of Food Technology, Arid Lands Cultivation Research Institute (ALCRI), City of Scientific Research and Technological Applications (SRTA-City), Alexandria, Egypt

**Keywords:** whey proteins, beetroot peels, antioxidant activity, proteins, functional beverages, betalains, bioactive components

## Abstract

**Introduction:**

Adequate protein and antioxidant intake are crucial for everyone, particularly athletes, to promote muscle performance and prevent muscle damage. Whey proteins are high-quality proteins with high digestibility and bioavailability; beetroot peels are an abundant antioxidant source.

**Methods:**

The present study was designated to develop a functional beverage based on mixing whey protein isolate (5%) with different concentrations of beetroot peel water extract (1, 2.5, and 5%) and flavored with strawberries puree (5%). In addition, we examined the stability of the physicochemical parameters and the bioactive components of the beverages during cold storage (4°C) for 14 days.

**Results and discussion:**

Whey protein isolates enriched the juices with stable protein content during the storage (4.65–4.69%). Besides, the extract revealed a concentration-dependent effect on the bioactive components, the antioxidant activity, and the microbial load of the juices; it distinguished the fresh juices by high betalains and nitrate content, 87.31–106.44 mg/L and 94.29–112.59 mg/L, respectively. Beverages with 2.5% peel extract (T2) had the preferable sensory attributes compared to control and other treatments. On day 0, phenolics and flavonoids increased in T2 by 44 and 31% compared to the control, which elevated the scavenging activity of the juice (T2) (*P* < 0.05). At the end of the storage period (14 days), phenolics and flavonoids of T2 recorded their lowest values, 26.23 and 21.75 mg/mL, respectively. However, they stood higher than phenolics (22.21 mg/mL) (*p* < 0.05) and flavonoids (18.36 mg/mL) (*p* > 0.05) of control. Similarly, betalains degraded by 45% to reach 47.46 mg/L in T2, which reduced the redness (a^*^) and increased the yellowness (b^*^) values.

**Conclusion:**

Consequently, whey/strawberry/beetroot peel (5: 5: 2.5 w/v/w) in d.H_2_O is a functional beverage that provides the body with a high-quality protein and a considerable amount of antioxidants.

## Introduction

Sufficient protein intake is vital for everyone (athletes and sedentary persons): Proteins increase the protein muscle synthesis, compensate for the amino acid loss (~50–60 g/70–90 kg) between the proteins synthesis and breaking down (1), and elevate the satiety feeling (2). Whey, the by-product of cheese manufacturing (3–5), is produced in significant amounts 10 liters/ kg cheese. Whey protein isolate (WPI) is one of the valuable products manufactured by removing water and lactose from the whey to valorize the whey and reduce its chemical and biochemical oxygen demand. WPI shares a protein content of up to 92% with desirable food technological properties such as high solubility, gelling, foaming, and emulsion formation (6). Whey is a rich source of high-quality proteins; it is rich in branched amino acids (leucine, isoleucine, and valine) that enhance muscle protein synthesis (7). Besides, whey is an abundant source of minerals; therefore, it is recommended as an isotonic beverage to compensate for the hydro electrolytic loss during exercising (5, 8).

Furthermore, adequate antioxidant intake participates in scavenging the reactive oxygen species (ROS) developed during practicing high-intense exercises or from exposure to environmental pollutants (9). Antioxidants obtained naturally from food matrices are more efficient in scavenging the ROS than purified antioxidant supplements, such as vitamin E (9). Hence, adding fruits and vegetables could evolve twin benefits in boosting the antioxidant activity of the products and enhancing their sensory properties. Beetroot (*Beta vulgaris* L.) belongs to the *Chenopodiaceae* family, a yellow or red-colored root. Red beetroot is the most commonly consumed worldwide (10). Beetroot wastes (peel, pomace, stem, and leaves) proved their functional and nutraceutical potential: They extended the shelf life of *Tilapia* fish fillets (11); enhanced the nutritional quality of orange juice (12), mayonnaise (13), and biscuits (14). In addition, beetroot wastes positively improved health by attenuating oxidative stress (12, 14, 15). Beetroot peel is one of the agricultural wastes that attracted more attention recently as it contains about 50% of the total antioxidant activity in the root (16): It is richer in betalains, mainly betacyanins (11), catechin, gallic acid, and syringic acid (10) than other beetroot parts. Accordingly, beetroot peels could have nutraceutical, antioxidant, and antimicrobial potential, denoting the high possibility of using beetroot peels in food products.

Therefore, utilizing the food by-products in developing an added-value beverage high in protein and antioxidants possesses many advantages. From the nutritious perspective, it participates in developing cost-effective, functional, and nutraceutical products (11, 12) based on sustainable food sources rich in bioactive components and nutrients (17). Moreover, it represents an extra income source from the economic perspective (18). Besides, it decreases the harmful effect on the environment by reducing pollution, greenhouse emissions (19), and biochemical and chemical oxygen demand of discarded food wastes (4, 6).

Accordingly, whey-based beverages enriched with plant by-products represent functional beverages for athletes that could reduce the need for other expensive protein sources and the oxidative stress induced by intensive exercises. In this context, we aimed to develop a mixture of high-protein and high-antioxidant beverages based on sustainable food waste flavored with strawberries. In addition, we determined the stability of the physicochemical parameters and bioactive components of the beverages during 14 days of storage in the refrigerator at 4°C.

## Materials and methods

### Materials

Beetroot and strawberries were procured from the local market in Alexandria, Egypt. Whey protein isolate (WPI) was brought from Burt Lewis Ingredients, LLC (United States), with a proximate composition depicted in Table 1.

**Table 1:** Proximate composition of whey protein isolate (WPI) and beetroot peels expressed on dry basis in (g/100 g).

	**WPI**	**Beetroot peels**
Moisture	6 ± 0.00^b^	8.8 ± 0.42^a^
Protein	91 ± 0.00^a^	7.45 ± 0.08^b^
Fat	3 ± 0.00^a^	1.46 ± 0.20^b^
Total carbohydrates	0 ± 0.00^b^	82.29 ± 0.25^a^

Data are expressed in means ± SD.

Means with different letters in the same row are statically different (*p* < 0.05).

### Solvents and reagents

Sodium carbonate, sodium hydroxide, sodium nitrate, aluminum chloride, methanol, and ethanol were obtained from Aljomhoria company, Alexandria, Egypt. Folin-Ciocalteu reagent, DPPH (2,2-diphenyl-1-picrylhydrazyl), ABTS (2, 2′-Azino-bis (3-ethylbenzothiazoline-6-sulfonic acid), salicylic acid, and phenolic standards were purchased from Merck, Darmstadt, Germany.

### Preparation of the plant materials

#### Strawberries puree

Strawberries were sorted and washed carefully to eliminate any undesired materials and plants. Good-shaped strawberries were pureed by a hand blender (Philips, HR1600/01, China); the puree was stored at −18 ± 2°C until added to beverages as a natural flavor due to its high acceptability among consumers in dairy products (20).

#### Water extraction of the bioactive components from beetroot peel

Fresh beetroot peels (with a proximate composition demonstrated in Table 1) were washed carefully under running tap water to remove any undesired materials before being cut into small pieces. Then, the peels were immersed in boiled water (100°C) (1:10 w/v) and fully covered with aluminum foil for 10 min. After stirring the mixture at room temperature for 3 h, the mixture was centrifuged at 3,000 rpm for 10 min at 20°C. Afterward, the collected supernatant was filtered, lyophilized by a vacuum freeze dryer (FDE 0350, Humanlab Inc., Bucheon-si, Gyeonggi-do, Korea), and stored at −18 **±** 2°C for further analysis and use as an additive (21).

### Characterization of beetroot peels water extract

#### Determination of total phenolics

Total phenolics of beetroot peel water extract were determined as described by Vodnar et al. (18). Briefly, 1 mL of Folin-Ciocalteu reagent (0.2 N) was mixed with 200 μL of peel water extract before adding 800 μL of NaCO_3_ (7.5%). After incubating the mixture at room temperature in the dark for 2 h, we measured the absorbance of the mixture at 760 nm with a spectrophotometer (Jenway 6405UV/VIS, Stone, Staffordshire, UK). The total phenolics of beetroot peels were expressed in mg/g gallic acid.

#### Determination of total flavonoids

To determine the total flavonoids, we followed the aluminum chloride method (12). A mixture of peel extract (1 mL), d.H_2_O (4 mL), and 5% sodium nitrate (300 μL) was incubated for 5 min before adding 300 μL of aluminum chloride (10%). After incubating the mixture for another 6 min, we added NaOH (2 mL) and completed the mixture to 10 mL with d.H_2_O. The absorbance of the samples was measured at 510 nm; the total flavonoids were expressed in mg/g catechin.

#### Determination of betalains content

We determined the betalains content in beetroot peel water extract spectrophotometrically by measuring the absorbance of the betacyanins at 353 nm and the betaxanthins at 438 nm (10). Betacyanins and betaxanthins content (mg/L) were calculated according to Equation (1); betalains were calculated according to Equation (2) and expressed in mg/L.


(1) $$\begin{array}{l} \text{Betacyanins/Betaxanthins }\left(\text{mg/L}\right)=\frac{\text{A}\times \text{DF}\times \text{MW}\times \text{V}}{\varepsilon \text{L}} \end{array}$$

Where (A) is the absorbance of the samples; (DF) is the dilution factor of the extract; (MW) is the molecular weight of the pigments (550 g/mol for betacyanins and 308 g/mol for betaxanthins); (V) is the volume of the extract expressed in mL. (*ε*) is the extinction coefficient of the pigments in water, 60,000 L/mol cm and 48,000 L/mol cm for betacyanins and betaxanthins, respectively, and (L) is the length of the cuvette path (1 cm).


(2) $$\begin{array}{l} \text{Betalains }\left(\text{mg/L}\right)=\text{Betacyanins}\left(\text{mg/L}\right)\\ \;+\text{Betaxanthins}\left(\text{mg/L}\right) \end{array}$$

#### HPLC analysis of the phenolics

To identify the total phenolics of beetroot peels, we injected 5 μL of the water extract into an HPLC (Agilent, Agilent 1260 series, Stevens Creek BLVD, San Jose, CA, USA). The phenolics were separated on an Eclipse C18 column (4.6 ×250 mm i.d., 5 μm) at 40°C. Water (A) and 0.05% trifluoroacetic acid in acetonitrile (B) were used as a mobile phase at a flow rate of 0.9 mL/min. The mobile phase was programmed consecutively in a linear gradient as follows: 0 min (82% A); 0–5 min (80% A); 5–8 min (60% A); 8–12 min (60% A); 12–15 min (82% A); 15–16 min (82% A) and 16–20 (82%A). The multi-wavelength detector was monitored at 280 nm.

#### Antioxidant activity

##### DPPH^•^ Scavenging activity

Briefly, 0.5 mL of a fresh DPPH^•^ in methanol (0.3 mM) was added to 0.5 mL of peel extract (at different concentrations). After incubating the mixture for 20 min at an ambient temperature (25°C), the absorbance of the mixture was measured at 517 nm (12, 22). The DPPH^•^ inhibition% was calculated as illustrated in Equation (3) to estimate the IC_50_ concentrations.


(3) $$\begin{array}{l} \text{\% Inhibition}=\frac{\text{absorbance of control }-\;absorbance\;of\;sample}{\text{absorbance of control}}\\ \;\times 100 \end{array}$$

##### ABTS^•^ scavenging activity

ABTS^•^ solution (7 mmol/l) was mixed with potassium persulfate (2.4 mmol/l) (1:1 v/v) and incubated in the dark for 12 h. The prepared reagent (1 mL) was then diluted with d.H_2_O (60 mL) to record an absorbance rate of 0.701 ± 0.01 at 734 nm. Afterward, the diluted reagent (4 mL) was added to 10 μL of the extract before being incubated for 6 min. The absorbance of the control and the samples was measured at 734 nm (23). ABTS^•^ scavenging activity was calculated from Equation (4) as follows:


(4) $$\begin{array}{l} \text{\%Inhibition}=\frac{\text{absorbance of control }-\text{ absorbance of sample}}{\text{absorbance of control}}\\ \;\times 100 \end{array}$$

### Nitrate content

Beetroot is an abundant nitrate source, indicating its advantages to human health and athletes.Accordingly, we determined the nitrate content of beetroot peels as described by Cataldo et al. (24). Briefly, 0.8 mL of freshly prepared salicylic acid in concentrated H_2_SO_4_ (5% w/v) was mixed with 0.2 mL of the extract and was left at room temperature for 20 min. Afterward, 19 mL of NaOH (2 N) was pipetted into the mixture to increase the pH to > 12. Before measuring the absorbance of the samples at 410 nm, we left the samples until being cooled down. To avoid the effect of the plant pigment, we corrected the absorbance of the samples by using a blank consisting of the extract (0.2 mL), concentrated H_2_SO_4_ (0.8 mL), and NaOH (19 mL) (without adding salicylic acid).

#### Antimicrobial effect of peel extract

The antimicrobial effect of beetroot peel water extract was determined against three bacterial strains by the Agar well diffusion method (25). One Gram-positive strain (*Staphylococcus aureus* NCTC 10788) and two Gram-negative strains (*Escherichia coli* BA 12296 and *Salmonella senftenebera* ATCC 8400) were obtained from the Genetic Engineering and Biotechnology Research Institute (GEBRI), Scientific Research and Technological Applications (SRTA-City), Borg Al-Arab, Alexandria, Egypt. The strains were incubated overnight at 37°C in a nutrient broth medium. Afterward, plate count agar medium (PCA) was poured into sterile Petri dishes containing 1 mL of (1 × 10^8^ CFU/mL) of each freshly grown bacterial strain. After mixing the medium and the inoculum, the plates were left to solidify. Four wells were created by a sterile cork borer (6 mm) in each plate; the wells were pipetted with 100 μL of the extract at concentrations of 0, 5, 10, and 15% (w/v). Then, the plates were left at 4°C for 30 min to ensure the diffusion of the extracts into the plate. After incubating the plates at 37°C for 18 h, the inhibition zone was measured and expressed in mm.

### Preparation of the whey-beetroot peel beverage

To prepare the whey-based beverage, we mixed 5% of whey protein isolate (91% protein), 5% of strawberries puree, and 10% of sugar in d.H_2_O at (45°C) to increase the solubility of the whey proteins (26). The beverage was enriched with beetroot peel extract at concentrations of 0, 1, 2.5, and 5% to prepare control, T1, T2, and T3 beverages, respectively (Table 2). The selection of the concentrations of the WPI (5%), strawberry puree (5%), and beetroot peel extract (1–5%) was based on our preliminary experiments. The prepared juices were pasteurized at 63°C for 30 min after homogenizing for 5 min. Afterward, the beverages were stored in the refrigerator at 4 ± 1°C; the samples were collected on days 0, 7, and 14 for further analysis.

**Table 2:** Ingredients of whey-beetroot peel beverages expressed in (%).

	**Control**	**T1**	**T2**	**T3**
WPI^*^	5	5	5	5
Strawberries puree	5	5	5	5
Sugar	10	10	10	10
Beetroot peel extract	0	1	2.5	5

* WPI, Whey protein isolate.

### Sensory evaluation

The sensory attributes of strawberry-whey beverages enriched with different concentrations of beetroot peel water extract were assessed by 20 panelists on day 0 (12 females and 8 males, 24–65 years old) and 15 panelists on days 7 and 14 (10 females and 5 males, 24–65 years old). The panelists were asked to evaluate each sensory characteristic of nine scores on the (1–9) Hedonic scale 1 extremely dislike; 9 extremely like (27). The beverages (4 ± 1°C) were left to rest at room temperature for 10 min before being served (28). The randomly codded beverages enriched with the peel extract (T1, T2, and T3) were served in transparent glasses after the control beverage to assess the acceptability of their color, odor, appearance, consistency, taste, flavor, and acidity.

### Physicochemical composition of the beverages

#### Total soluble solids

TSS of the beverages was measured in°Brix by a digital refractometer NR101.

#### pH

The pH of the juices was determined at 25°C by a pH meter (Adwa, AD1030, Szeged, Hungary).

#### Total titratable acidity (TTA)

To determine the (TTA), we titrated the diluted beverages (1:10 v/v) with 0.1N NaOH until we obtained a pH value of 8.2 (10). The total acidity was expressed as (g lactic acid/100 mL) according to Equation (5).


(5) $$\begin{array}{l} \text{\%Acidity}=\frac{\text{V }\times \text{ N }\times \;0.09\;\times 100}{\text{w}} \end{array}$$

Where (V) is mL of NaOH consumed during the titration; (N) is the normality of NaOH; (W) is the weight of the sample.

#### TSS/TTA ratio

The ratio is calculated by dividing the total soluble solids (TSS) of the beverage by its total titratable acidity (TTA). Hence, we could theoretically predict the acceptability of the beverage (12).

#### Viscosity

The viscosity of the beverages was determined at room temperature by a viscometer (STS-2011, Spain).

#### Color

The color of the beverages was measured by a colorimeter (HunterLab EasyMatch QC, Reston, Virginia, USA); the data were expressed in L^*^ (brightness/darkness), a^*^ (redness/greenness), and b^*^ (yellowness/blueness).

#### Total protein content

The total protein of the samples was estimated by the Kjeldahl method (29).

### Bioactive components of the beverages

The bioactive components of the beverages during the storage period were extracted according to Silveira et al. (30). Briefly, 0.5 mL of the beverages were mixed with 9.5 mL of acetone (70%) before centrifuging at 12,000 rpm for 15 min. The supernatants were collected to estimate the total phenolics, the total flavonoids, the betalains, and the DPPH^•^ scavenging activity of the prepared beverages during storage time (0, 7, and 14 days) were estimated as described previously in Sections 2.3.1, 2.3.2., 2.3.3., and 2.3.5.1, respectively.

### Beverages' shelf-life and microbial load

The beverages were well mixed by a vortex (WiseMix VM-10, witeg Labortechnik, GmbH, Wertheim, Germany) before being subjected to a 10-folds serial dilution. Afterward, to enumerate the total plate, coliform, and yeasts and molds, 1 mL of the suitable dilutions was transferred into Petri dishes filled with plate count agar (PCA), Violet red bile agar (VRBA), and Rose bengal agar-based media, (LAB M Ltd, Lancashire, United Kingdom), respectively. After incubating the plates at 37°C for 24 h, the grown colonies were counted and expressed as Log^10^ CFU/mL (31).

### Statistical analysis

The statistical analysis in the present research was performed by the IBM SPSS program 25, Armonk, New York, United States. The data were analyzed by one and two-way ANOVA tests. The means were compared by Duncan's test at a confidence level of 95% (*p* < 0.05); the obtained data were expressed in mean ± standard deviation (SD).

## Results

### Characteristics of beetroot peel extract

**Phenolics and flavonoids:** Beetroot peels are a rich source of phenolics and flavonoids (Table 3). The total phenolics of beetroot peel water extract was 83.17 ± 9.03 mg/g; the flavonoids represent nearly a quartile of the total phenolics in the beetroot peel water extract, 21.70 ± 2.18 mg/g.

**Table 3:** Characteristics of beetroot peel water extract.

**Bioactive components**
Total phenolics (mg/g)	83.17 ± 9.03
Total flavonoids (mg/g)	21.70 ± 2.18
Betacyanins (mg/L)	236.53 ± 10.82
Betaxanthins (mg/L)	155.54 ± 0.36
Betalains (mg/L)	392.07 ± 10.55
Nitrate (mg/L)	1528.60 ± 73.18
**Phenolic profile (μL/mL)**	
Chlorogenic acid	23.12
Gallic acid	7.08
Syringic acid	2.04
Caffeic acid	0.37
Ferulic acid	0.52
**Antioxidant activity (μL/mL)**	
DPPH IC_50_	125.12 ± 3.05
ABTS IC_50_	33.45 ± 0.10
**Antimicrobial activity (mm)**	
*Staphylococcus aureus NCTC* 10788	ND
*Escherichia coli BA* 12296	13 ± 0.71
*Salmonella senftenebera* ATCC 8400	12 ± 0.85

ND, Not detected.

**Betalains:** Beetroot peels contain a high concentration of betalains, 392.07 ± 10.55 mg/L (Table 3). The betacyanins represent about 60% of the dye (236.53 ± 10.82 mg/L); it was 1.5 times higher than the betaxanthins content.

**Phenolic profile:** In the water extract of beetroot peels, we detected chlorogenic acid in high concentrations (Table 3; Supplementary material). Chlorogenic acid represented 70% of the detected phenolics, followed by 21% of gallic acid, 6% of syringic acid, 2% of ferulic acid, and 1% of caffeic acid.

**Antioxidant activity:** Due to the high concentrations of phenolic compounds and betalains, beetroot peel water extract revealed a high antioxidant activity. Small amounts of the extract were able to scavenge the DPPH^•^ and the ABTS^•^ radicals, as 125.12 ± 3.05 μL/mL and 33.45 ± 0.10 μL/mL of the peel water extract were able to reduce 50% of the DPPH^•^ and the ABTS^•^ radicals, respectively (Table 3).

**Nitrate content:** Beetroot peels exhibited a considerable nitrate content (Table 3), 1528.60 ± 73.18 mg/L.

**Antimicrobial effect:** Unlike the antioxidant ability, beetroot peel water extract displayed an intermediate antimicrobial effect. Although the extract did not reveal any inhibitory effect against the Gram-positive *Staphylococcus aureus* at 15 mg/mL, the extract showed an intermediate inhibitory effect against the Gram-negative *Escherichia coli* and *Salmonella senftenebera*, with inhibition zones of 13 and 12 mm, respectively (Table 3).

### Sensory attributes of whey-strawberry beverage with beetroot peel extract

**Figure 1:**
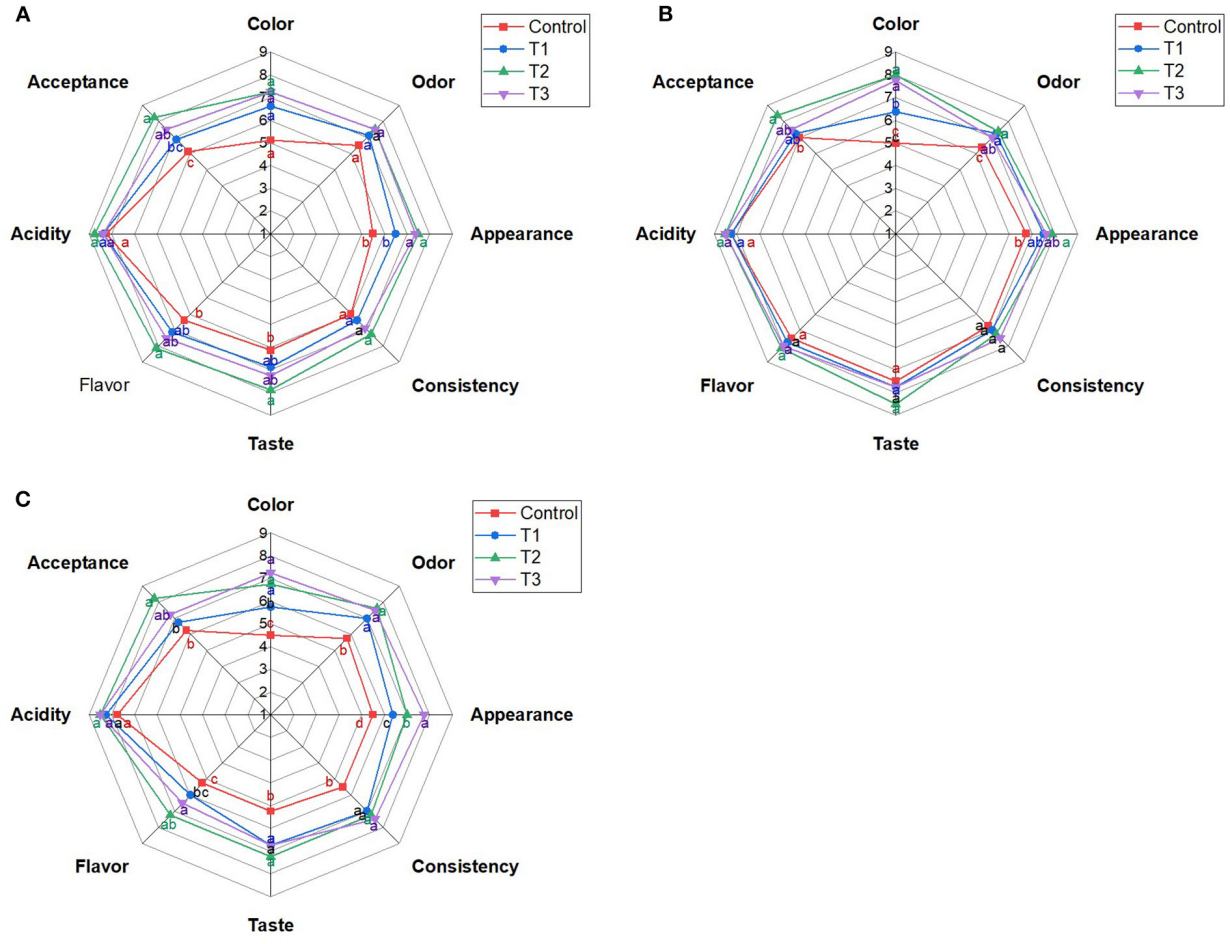
Sensory attributes of whey/strawberry beverages enriched with different concentrations of beetroot peel water extract on day 0 **(A)**, day 7 **(B)**, and day 14 **(C)** of storage at 4°C. Means with different letters (a–d) for each sensory attribute are statistically different (*p* < 0.05).

Mixing strawberry puree (5%) with the whey beverage developed an acceptable control beverage, with 6.13 and 6.38 degrees for taste and flavor, respectively, on day 0 (Figure 1A). Adding beetroot peel water extract at concentrations of 1, 2.5, and 5% to the whey/strawberry beverage strengthens the sensory attributes of the beverage: The color, appearance, taste, flavor, and total acceptance of the juices (T1, T2, and T3) were improved (*p* < 0.05) after adding the beetroot peel extract compared to the control beverage on day 0. Nevertheless, T2 revealed the highest acceptability among the other blends, mainly taste and flavor (Figure 1A).

The sensory test on day 7 revealed minor changes in the sensory attributes of the beverages compared to day 0; T2 remained the most preferred formula compared to the other juices (P < 0.05; Figure 1B). On the other hand, taste and flavor acceptability changed noticeably on day 14 compared to day 0 (Figure 1C). Generally, T2 had the highest acceptability among other beverages during storage (*p* < 0.05). Storage of the beverages for 14 days declined the acceptability of color, taste, and flavor compared to the freshly prepared beverages (*p* < 0.05). While the odor, appearance, consistency, acidity, and total acceptability were insignificantly affected.

### Physicochemical properties of the beverages

**Figure 2:**
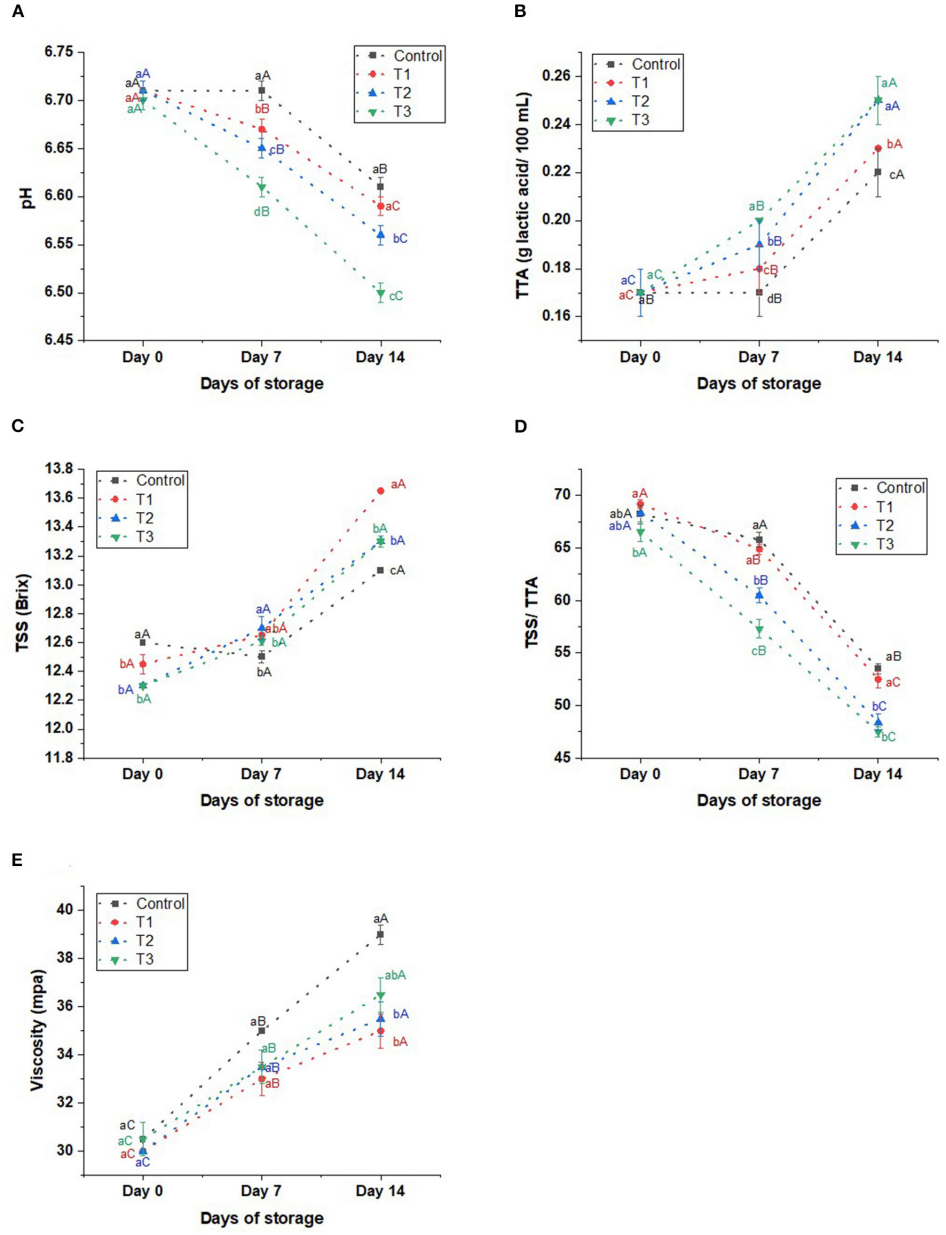
**(A)** pH, **(B)** total titratable acidity (TTA) in g lactic acid/100 mL, **(C)** total soluble solids (TSS) in Brix **(D)** TSSITAA ratio, and **(E)** viscosity in mpa of control beverage and beverages enriched with beetroot peel extracts at different concentrations. Means with different lowercase letters (a–d) on the same storage day are statistically different (*p* < 0.05). Means with different uppercase letters (A–C) for each treatment during the different storage days are statistically different (*p* < 0.05).

**pH:** The pH of the beverages tended to be neutral, in a range of 6.50–6.71, during the storage period (14 days) (Figure 2A). However, The extract gradually reduced the pH of treatments in a dose-dependent manner (*p* < 0.05) compared to the control on 7 and 14 days of storage. We also noticed that the pH of all samples declined (*p* < 0.05) with prolonged storage.

**Total titratable acidity (TTA):** Peel extract did not affect the acidity of the fresh beverage (T1, T2, and T3) (*p* > 0.05) compared to the control, 0.17% (Figure 2B). However, on days 7 and 14, we noticed that the acidity of the juices (T1, T2, and T3) was increased gradually by increasing the concentration of the extract compared to the control. Besides, the storage participates in elevating (*p* < 0.05) the acidity of the products by 30–47% on day 14 of the storage, 0.22–0.25 g lactic acid/100 mL in control, T1, T2, and T3.

**Total soluble solids (TSS):** The extract did not affect the TSS of the beverage, as the changes among treatments (T1, T2, and T3) and the control sample were insignificant, being in a range of 12.30–12.60 on day 0, 12.50–12.70 on day 7, and 13.10–13.65 on day 14 (Figure 2C). However, the TSS of all beverages increased significantly by the end of the storage.

**TSS/TTA ratio:** On day 0, T1, T2, and T3 shared TSS/TTA ratio as the control, in a range of 68.20–69.17. The TSS/TTA was reduced significantly by increasing the peel extract compared to the control on days 7 and 14: The higher concentration of the extract, the lower the acceptability (Figure 2D). The TSS/TTA ratio of the beverages was reduced significantly on day 14 by 21.57, 24.10, 29.20, and 28.61% in control, T1, T2, and T3, respectively.

**Viscosity:** The extract insignificantly affected the viscosity of T1, T2, and T3 compared to the control (30–30.50 mpa) on day 0 (Figure 2E). During the storage, the viscosity of the control juice increased sharply (*p* < 0.05) to reach 39 mpa on day 14. Similarly, the viscosity of the treatments increased during the storage period but at lower rates than in control.

**Figure 3:**
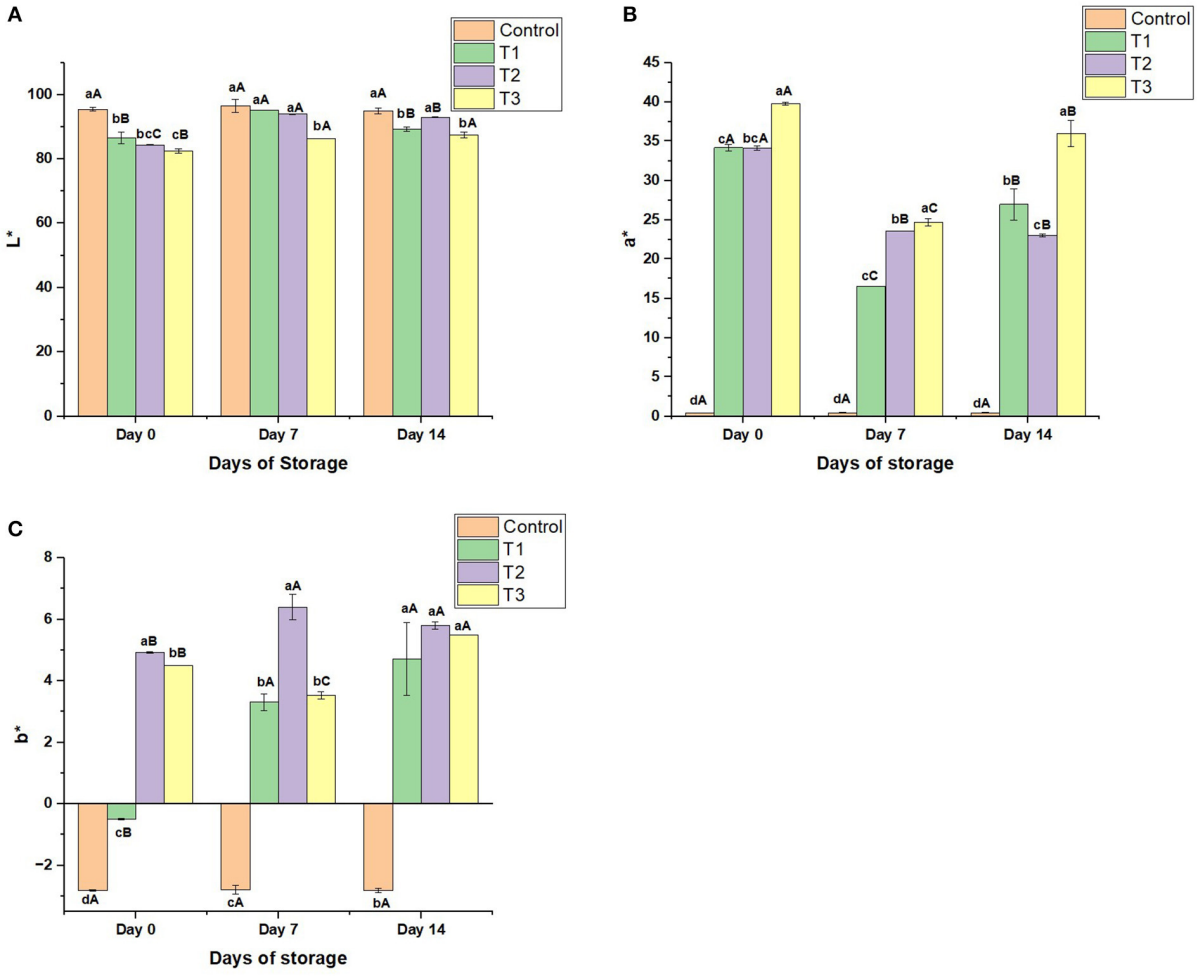
**(A)** Lightness (L*) values, **(B)** Redness/greenness (a*) values, and **(C)** Blueness/yellowness (b*) values of control beverage and beverages enriched with beetroot peel extracts at different concentrations during 14 days of storage. Means with different lowercase letters (a–d) on the same storage day are statistically different (*p* < 0.05). Means with different uppercase letters (A–C) for each treatment during the different storage days are statistically different (*p* < 0.05).

**Color:** As noticed in Figures 3A–C, the control sample was white-colored with a slight redness due to the small concentration of strawberries (5%) in the beverage. On the other hand, T1, T2, and T3 on day 0 were white-colored with moderate redness. The redness values increased from 34.1 to 39.75 (on day 0) in the treatments by increasing the peel concentration (Figure 3B). During the storage, the color of the control sample revealed high stability; the change in the color was not noticeable, with an (a^*^) range of 0.42–0.45. On the opposite, the treatments exhibited moderate redness stability during storage; the change in the color of the beverages enriched with the peel extract was perceptible. The redness of the beverages on day 7 was reduced by 22.26% in T1, 27.75% in T2, and 40.78% in T3 compared to day 0. On the other hand, the yellowness of the treatments increased to reach 6.40 on day 7 before decreasing slightly to 5.80 on day 14 (Figure 3C).

**Figure 4:**
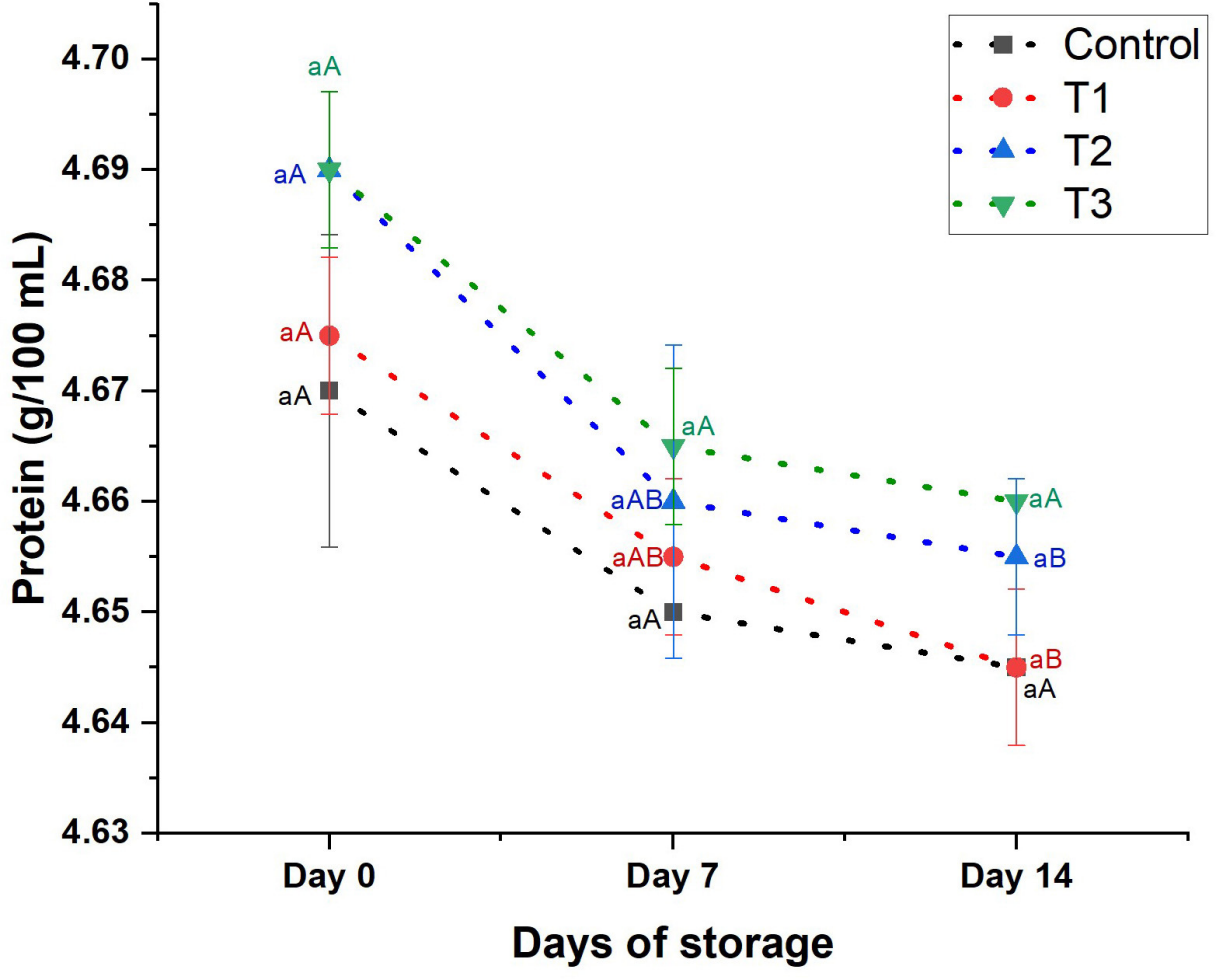
Protein content (g/100 mL) of control beverage and beverages enriched with beetroot peel extracts at different concentrations. Means with different lowercase letters (a–d) on the same storage day are statistically different (*p* < 0.05). Means with different uppercase letters (A–C) for each treatment during the different storage days are statistically different (*p* < 0.05).

**Protein content:** Figure 4 depicts the protein content of juices during the storage period (14 days). The protein content of the beverages (control, T1, T2, and T3) revealed insignificant changes, being 4.67–4.69 on day 0, 4.65–4.67 on day 7, and 5.65–4.66 on day 14. The protein content of the juices was slightly reduced (*p* < 0.05) on day 14 by 0.54, 0.64, 0.64, and 0.53 in control, T1, T2, and T3, respectively.

### Bioactive components of the beverages

**Figure 5:**
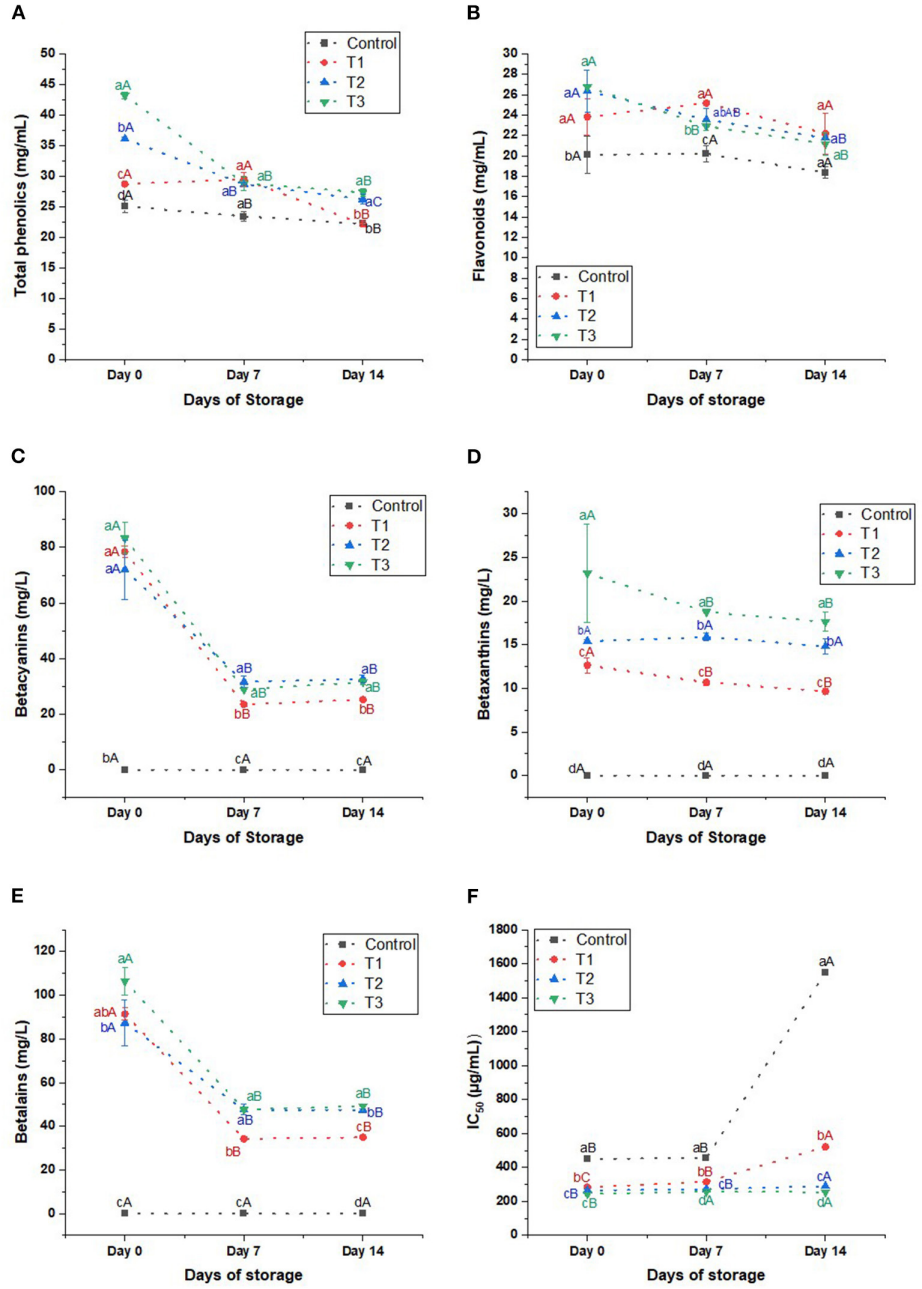
**(A)** Total phenolics (mg/mL), **(B)** flavonoids (mg/mL), **(C)** betacyanins (mg/L), **(D)** betaxanthins (mg/L), **(E)** betalains (mg/L), and **(F)** IC_50_ (μL/mL) of control beverage and beverages enriched with beetroot peel extracts at different concentrations. Means with different lowercase letters (a–d) on the same storage day are statistically different (*p* < 0.05). Means with different uppercase letters (A–C) for each treatment during the different storage days are statistically different (*p* < 0.05).

**Phenolics**: The total phenolics of the beverages are elevated significantly by increasing the percentage of the extract (Figure 5A). The phenolics of T2 and T3 were 1.4 and 1.7 higher than the control, 36.12 and 43.21 mg/g, respectively, on day 0. Likewise, on days 7 and 14, the phenolics of the treatments, particularly T2 and T3, were higher than the control. On day 14, we noticed a significant reduction in the phenolics by 11.44, 22.80, 27.30, and 36.70% compared to day 0 in control, T1, T2, and T3, respectively. Despite the reduction in the phenolics of the beverages during the storage, T2 and T3 had more abundant phenolics than the control.

**Flavonoids:** The total flavonoids increased in the beverages by increasing the percentage of the peel extract (Figure 5B). On day 0, beetroot peel did not affect the flavonoid content of T1 (*p* > 0.05) compared to the control. Still, by increasing the percentage of the beetroot peels in T2 and T3, the total flavonoids increased significantly by 31 and 33%, respectively. On day 7, the flavonoids of T1, T2, and T3 were higher than the control (*p* < 0.05). But on day 14, the flavonoids of the treatments were as control. As the storage days were prolonged, the flavonoids of the control and T1 insignificantly reduced by 8.7 and 6.8%, respectively. On the other hand, the flavonoids of T2 and T3 significantly declined on day 14 by 17.5 and 20.93%, respectively, compared to day 0.

**Betalains:** We did not detect betalains in the control beverage. But it was detected in the juices enriched with the peel extract in considerable amounts. The betalains content on day 0 increased by increasing the peel extract concentration to reach 106.44 ± 6.46 mg/L in T3 (Figure 5E). However, we noticed that the pigment was unstable during the storage period as the betalains degraded sharply (*p* < 0.05) after 7 days of storage to reach 62.6, 45.54, and 55.27 mg/L in T1, T2, and T3, respectively. But the pigment stood stable afterward until day 14. As noticed, the betalains content correlated to the betacyanins content of the beverages. The betacyanins in juices increased significantly by increasing the peel extract, ranging from 78.47 ± 1.94 to 83.28 ± 5.64 mg/L on day 0 of the storage (Figure 5C). However, it decreased sharply (*p* < 0.05) after 7 days of storage by 54.62–70%, but it stood stable until the 14th day of storage. On the other hand, betaxanthins experienced a minor significant reduction in T1 and T3 and an insignificant decline in T2 (Figure 5D).

**Antioxidant activity:** Generally, beverages enriched with peel extract revealed a higher antioxidant activity than the control (Figure 5F). T2 and T3 had the highest scavenging activity than the other blends: On day 0, 264.32, and 242.88 μL/mL of T2 and T3, respectively, were able to scavenge 50% of the DPPH^•^ radicals. Expectedly, the antioxidant activity of the beverages was reduced significantly during storage. However, we noticed that the antioxidant activity of treatments was 3–6 times higher than the control on day 14 compared to 1.5–1.9 times higher than the control on day 0.

**Figure 6:**
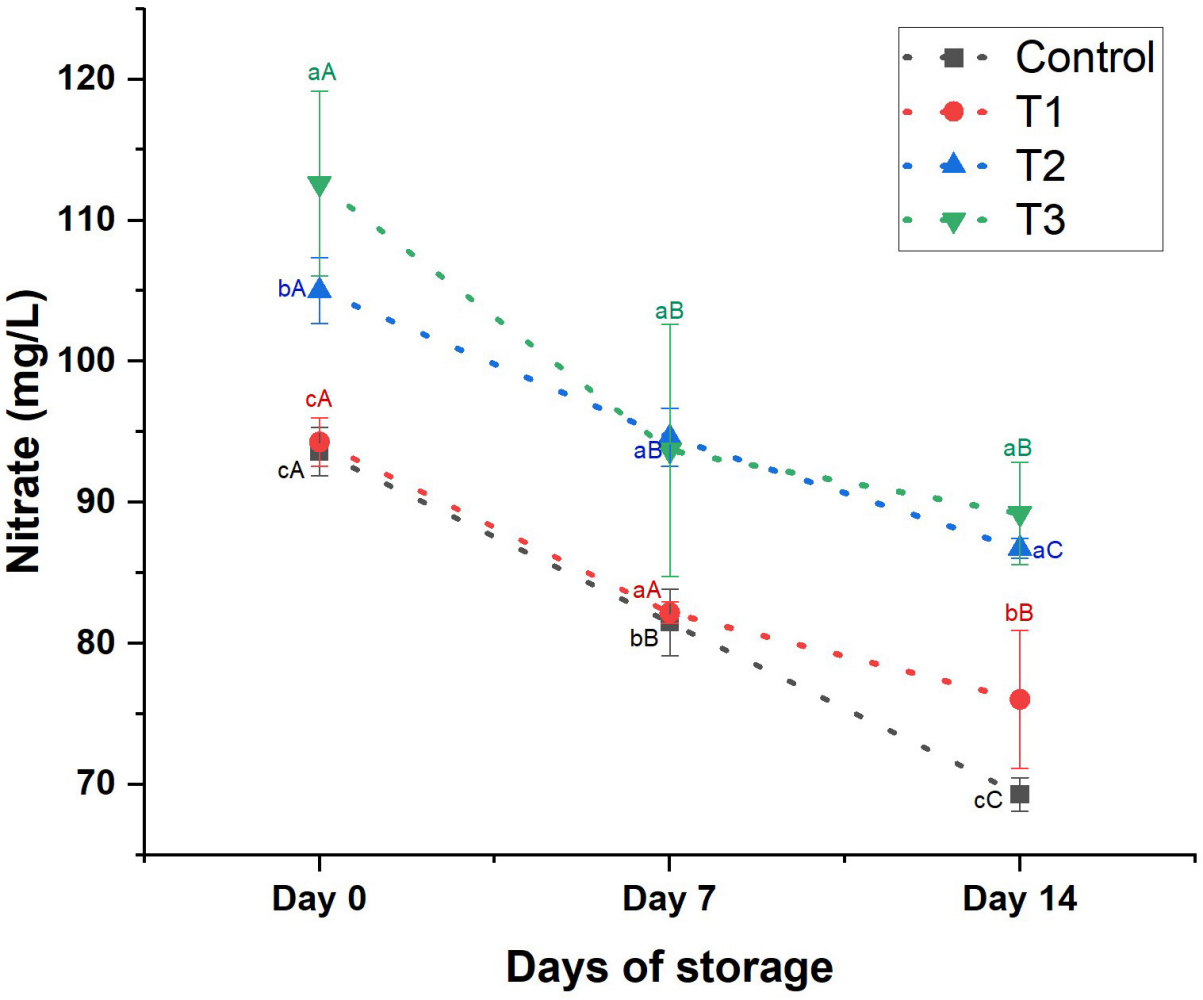
Nitrate content (mg/L) of control beverage and beverages enriched with beetroot peel extracts at different concentrations. Means with different lowercase letters (a–d) on the same storage day are statistically different (*p* < 0.05). Means with different uppercase letters (A–C) for each treatment during the different storage days are statistically different (*p* < 0.05).

**Nitrate content:** Nitrate was detected in low concentrations in the control juice (93.60 ± 1.71 mg/L). Adding beetroot peel extract increased the nitrate content of the treatments in a concentration-dependent manner (*p* < 0.05; Figure 6). T3 contained the highest nitrate content (112.59 ± 6.56 mg/L) compared to the other treatments. After 7 days of storage, we noticed a slight reduction (*p* > 0.05) in the nitrate content of T1; however, the nitrate content of the control, T2, and T3 declined significantly by 13, 10, and 17%, respectively. While on day 14, nitrate sharply dropped (*p* < 0.05) by 26% in control, 20% in T1, 17% in T2, and 21% in T3.

### Microbiological examination

Table 4 illustrates the total count of bacterial growth, yeasts and fungi, and coliform growth in juices with different concentrations of beetroot peel extract during 14 days of storage at 4°C. We could not detect microbial growth in all beverages during the storage time up to the 7th day. However, the microorganisms started to grow on day 14 in all juices, which was associated with the peel percentage as the growth reduced (*p* < 0.05) by increasing the peel percentage. T3 recorded the lowest microbial growth (1.87 × 10^1^), followed by T2 (3.47 × 10^1^) and T1 (25.00 × 10^1^). Beetroot peel extract inhibited microbial growth by nearly 95, 92, and 45% in T3, T2, and T1, respectively, compared to the control.

**Table 4:** Microbial growth of whey/strawberry beverage enriched with different concentrations of beetroot peel water extract during storage period.

	**Control**	**T1**	**T2**	**T3**
**Total count**				
**Day 0**	ND	ND	ND	ND
**Day 7**	ND	ND	ND	ND
**Day 14**	45.67 ± 15.43^aA^	25.00 ± 4.44^bA^	3.47 ± 0.74^cA^	1.87 ± 0.11^dA^
**Yeasts and molds**				
**Day 0**	ND	ND	ND	ND
**Day 7**	ND	ND	ND	ND
**Day 14**	39 ± 12.77^aA^	9.63 ± 0.71^bA^	5.50 ± 0.56^bA^	3.23 ± 0.25^bA^
**Coliform**				
**Day 0**	ND	ND	ND	ND
**Day 7**	ND	ND	ND	ND
**Day 14**	ND	ND	ND	ND

ND, Not detected.

Data are expressed in means of Log^10^ CFU/mL ± SD.

Means with different lowercase letters in the same row are statically different (*p* < 0.05).

Means with an uppercase letter in the same column indicate statical differences during the storage (*p* < 0.05).

Similarly, beetroot peel extract revealed a potent anti-fungal effect. It significantly reduced the fungal colonies' growth compared to the control in a concentration-dependent effect. On day 14, the fungi began to arise in the juices; however, the extract inhibited the fungal growth (*p* < 0.05) by 92, 86, and 75% in T3, T2, and T1, respectively, compared to the control.

On the other hand, no coliform colonies were detected in the beverages after manufacturing or during storage for 14 days.

## Discussions

Previous studies developed whey-based beverages as a high-quality protein source to enhance muscle performance and prevent protein deficiency. Few studies focused on boosting the antioxidant capacity of whey beverages by natural sources of antioxidants to scavenge the ROS developed during intensive exercise. Athletes depend on purified antioxidant supplements such as vitamin E, proving its frail scavenging activity of ROS. Beetroot peels are a sustainable antioxidant source, albeit underutilized; to our knowledge, no previous studies investigated the effect of adding beetroot peel extract to whey-based beverages. Therefore, we designated a whey beverage enriched with beetroot peel water extract as a natural antioxidant source. The whey provided the juices with a stable protein content during the storage (14 days) at 4°C. Besides, the extract distinguished the juices by betalains; the phenolics and flavonoids increased in a concentration-dependent manner, boosting the antioxidant activity of the juices (*p* < 0.05).

Beetroot peels are rich in bioactive components, boosting the extract's antioxidant and antimicrobial effect. Betalains are a water-soluble nitrogenous pigment (16) found in considerable amounts in peel water extract. Betacyanins represent 60% of the extracted betalains, distinguishing the extract by a peculiar red color. Besides, the phenolics and flavonoids in the peel extract were 10 and 5 times higher than that of Abdo et al. (10) and John et al. (32), respectively, with high concentrations of chlorogenic and gallic acids. Hence, peel water extract shared a high scavenging activity. Since beetroot is an abundant nitrate source (10), we detected 1528.60 ± 73.18 mg/L of nitrate in the water peel extract, showing beneficial effects on human health (33). Accordingly, the peel water extract inhibited the growth of *Escherichia coli* and *Salmonella senftenebera* but did not inhibit the growth of *Staphylococcus aureus*. Conversely, beetroot peel methanol extract (32) intermediately inhibited the *Staphylococcus aureus* growth. In this regard, beetroot peel extract is a sustainable and inexpensive antioxidant additive.

Mixing WPI (5%) with strawberry puree (5%) and beetroot peel water extract (1–5%) resulted in beverages with preferable sensory attributes, whereas T2 (2.5% extract) had the highest acceptability among all beverages. The low concentration of strawberry (5%) enhanced the flavor of the juices without affecting the neutral nature of the WPI; therefore, all juices exhibited neutral pH. Our finding agreed with Janiaski et al. (20), reporting that strawberries did not affect the neural pH of the whey beverage. Besides, the juices had high TSS/TTA ratios, reflecting their highest acceptability (12). The heightened redness of treatments resulted from the peel extract's betalains; we ignored the effect of the strawberries' anthocyanin because strawberry was equally added to all beverages.

The functional beverages were high in proteins and antioxidants. Whey resulted in a high-protein beverage (4.67–4.95%); beetroot peel extract increased the bioactive components in a concentration-dependent manner. Phenolics and flavonoids of treatments were in ranges of 28.72–43.21 and 23.83–26.76 mg/mL, respectively, which are higher than the control's phenolics (25.08 mg/mL) and flavonoids (20.12 mg/mL). Similarly, jabuticaba peel extract boosted the phenolic content of whey beverages (260 mg/L) (8). Besides, the extract boosted the beverages with betalain (91.40–106.44 mg/L). Consequently, the high antioxidant activity of the treated juices was strongly correlated to betalains (*r* = 0.99), flavonoids (*r* = 0.97), and total phenolics (*r* = 0.83) from the extract. Besides, the extract evolved the nitrate in the beverages but in a limited concentration, 94.29–112.59 mg/L, enhances blood flow to the muscles and improves the muscle's contracting and relaxation process. It also raises the blood flow, lowering blood pressure (34) and reducing cardiovascular disease (33). Accordingly, enriching the beverage with beetroot peel could benefit the athletes tremendously.

The sensory attributes of the juices are influenced by the storage period (14 days), as color, flavor, and taste highly influence the consumers' acceptability (35). However, T2 beverage remained the preferable beverage compared to other juices. Storage insignificantly affected the protein of juices; however, it significantly influenced the beverages' bioactive components, antioxidant activity, and physical properties. Betalains were the most sensitive component: After 7 days, betalains decreased by 46–63%, but it regenerated on day 14 by 0.19–3%, considerably influenced by betacyanins degradation and regeneration. Our finding was consistent with Kujala et al. (16). Betalains degraded into yellow-colored betalamic and isobetalamic acids (36), influencing the physical properties of the beverages: pH was reduced, whereas acidity and yellowness (b^*^) were increased (*p* < 0.05). The increase in the acidity declined the aggregation of the whey proteins, reducing the viscosity of the treatments (35–36.5 mpa) compared to the control (39 ± 0.41 mpa) during prolonged storage. Likewise, the nitrate gradually declined during storage; our result agreed with Corleto et al. (37), attributing the decline to nitrate reduction into nitrite. Although phenolics and flavonoids were significantly reduced during storage, phenolics were higher than the control, but flavonoids reached values as the control (*p* > 0.05). Accordingly, the antioxidant activity declined primarily because of the drop in betalains (*r* = 0.98) and phenolics (*r* = 0.88). However, the antioxidant activity of the treatments was 2–3 folds the antioxidant activity of the control during storage, possibly because the beetroot's bioactive components degraded into ingredients that potentiated the antioxidant activity of the treatments.

The absence of coliform in fresh and stored beverages indicates high hygiene practices during manufacturing and handling (38). The pasteurization inhibited microbial growth until day 7 of the storage. But on day 14, the bacteria and fungi were detected in treatments and control, albeit the extract declined the microbial load of the treatments in a concentration-dependent manner due to its antimicrobial effect. The extract potently inhibited bacterial growth compared to the fungal: It inhibited bacterial growth in T3 by 95% compared to 92% of fungal growth inhibition. Since beverages with 4.87–5.17 log CFU/mL of aerobic mesophilic bacteria and no coliform growth are safe to consume (39), T2 and T3, sharing 3.47 and 1.87 log CFU/mL on day 14, are considered safe for use for up to 14 days when stored at 4°C.

One of the limitations of the present study was the adequate antimicrobial characterization of the extract. Using a small concentration (0–15 mg/g) of the extract and excluding other pathogens from the experiment due to their frail activation ability limited the antimicrobial profile of the extract. Besides, we identified the phenolics by HPLC by one wavelength (280 nm) that affected flavonoid identification. Although we provided data about the chemical composition of the functional beverage and its stability during cold storage, further researches are crucial to proving the functionality of the Juice. One of the approaches to fulfill this purpose is examining the bioactive components' bioavailability of the beverage, investigating the effect of juices as an isotonic beverage, and studying the beverage's ability to enhance oxidative balance during and after exercising.

## Conclusion

Briefly, this study investigated the ability to develop a high protein and antioxidant beverage based on sustainable food by-products. Mixing WPI (5%) and beetroot peel water extract (1, 2.5, and 5%) with strawberry puree (5%) resulted in a neutral red-colored beverage with high acceptability, particularly T2 (with 2.5% peel extract). Strawberries enhanced the beverages' flavor; WPI resulted in beverages with a significant protein content, 4.67–4.69 g/100 mL. The extract enriched the beverages with betalains (91.4–106.44 mg/L) and nitrates (94.29–112.59 mg/L). From the extract, betalains and phenolics (chlorogenic, gallic, and syringic acids) potentiated the beverages' antioxidant activity in a concentration-dependent manner as 264.32 (T2) and 242.88 μL/mL (T3) scavenged 50% of the DPPH^•^. During cold storage (4°C) for 14 days, phenolics, flavonoids, nitrates, and betalains declined significantly, reducing the antioxidant activity of the beverages. However, the beverages' antioxidant activity was 3–6 times higher than the control. Besides, the extract extended the shelf-life of T2 and T3, with an acceptable aerobic growth of 3.47 and 1.87 log^10^ CFU, respectively. Consequently, the developed beverage could be a suitable and safe source of protein and antioxidants for individuals, particularly athletes, that could effectively benefit health.

## Data availability statement

The original contributions presented in the study are included in the article/Supplementary material, further inquiries can be directed to the corresponding author.

## Author contributions

EA: conceptualization and methodology, formal analysis, writing—original draft, and visualization. HM and MA: investigation and resources. OS and MG: validation and writing—review and editing. All authors have read and agreed to the published version of the manuscript.
