# Supplementary material for: Valorization of whey proteins and beetroot peels to develop a functional beverage high in proteins and antioxidants
Source: Front Nutr. 2022 Dec 14;9:984891. doi: 10.3389/fnut.2022.984891 (PMC9795000; doi:10.3389/fnut.2022.984891)
Supplement: Supplementary file 1 [file Data_Sheet_1.PDF]

Data File C:\CHEM32\1\DATA\PP 6-7-2022 2022-07-06 14-12-40\007-0701.D
Sample Name: Peel

===

Acq. Operator : SYSTEM   Seq. Line : 7
Acq. Instrument : hplc -2   Location : Vial 7
Injection Date : 7/6/2022 4:50:37 PM   Inj : 1
Inj Volume : 5.000 µl
Acq. Method : C:\CHEM32\1\DATA\PP 6-7-2022 2022-07-06 14-12-40\POLYPHENOL 2017.M
Last changed : 7/6/2022 2:12:42 PM by SYSTEM
Analysis Method : C:\CHEM32\1\DATA\PP 6-7-2022 2022-07-06 14-12-40\POLYPHENOL 2017.M (Sequence Method)
Last changed : 8/2/2022 11:46:29 AM by SYSTEM
(modified after loading)
Additional Info : Peak(s) manually integrated

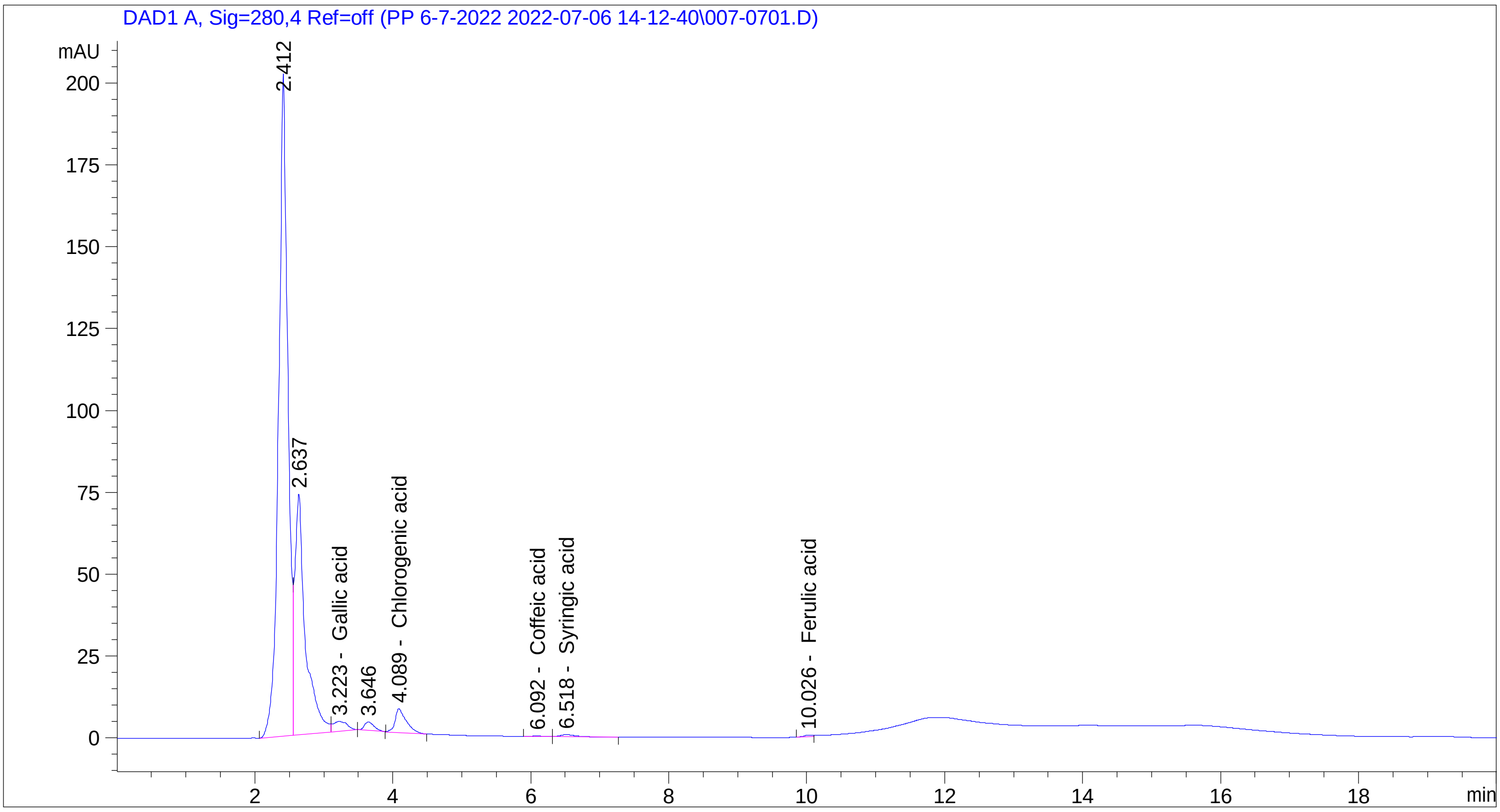

===

Area Percent Report

===

Sorted By : Signal
Calib. Data Modified : 7/6/2022 5:11:25 PM
Multiplier : 1.0000
Dilution : 1.0000
Use Multiplier & Dilution Factor with ISTDs

Signal 1: DAD1 A, Sig=280,4 Ref=off

Peak #	RetTime [min]	Type	Width [min]	Area [mAU*s]	Area %	Name
1	2.412	BV	0.1253	1896.68494	66.4850	?
2	2.637	VV	0.1471	788.66925	27.6454	?
3	3.223	VB	0.1944	44.05062	1.5441	Gallic acid
4	3.646	BB	0.1544	26.05475	0.9133	?
5	4.089	BB	0.1577	82.10516	2.8781	Chlorogenic acid

Peak #	RetTime [min]	Type	Width [min]	Area [mAU*s]	Area %	Name
6	4.588		0.0000	0.00000	0.0000	Catechin
7	5.580		0.0000	0.00000	0.0000	Methyl gallate
8	6.092	BV	0.2269	2.17931	0.0764	Coffeic acid
9	6.518	VB	0.2277	9.78779	0.3431	Syringic acid
10	6.749		0.0000	0.00000	0.0000	Pyro catechol
11	7.969		0.0000	0.00000	0.0000	Rutin
12	8.791		0.0000	0.00000	0.0000	Ellagic acid
13	9.096		0.0000	0.00000	0.0000	Coumaric acid
14	9.742		0.0000	0.00000	0.0000	Vanillin
15	10.026	BV	0.1178	3.27159	0.1147	Ferulic acid
16	10.476		0.0000	0.00000	0.0000	Naringenin
17	12.235		0.0000	0.00000	0.0000	Daidzein
18	12.713		0.0000	0.00000	0.0000	Querectin
19	14.023		0.0000	0.00000	0.0000	Cinnamic acid
20	14.476		0.0000	0.00000	0.0000	Apigenin
21	14.979		0.0000	0.00000	0.0000	Kaempferol
22	15.568		0.0000	0.00000	0.0000	Hesperetin

Totals : 2852.80341

7 Warnings or Errors :

Warning : Calibration warnings (see calibration table listing)
Warning : Calibrated compound(s) not found
Warning : Invalid calibration curve, (Gallic acid)
Warning : Invalid calibration curve, (Chlorogenic acid)
Warning : Invalid calibration curve, (Coffeic acid)
Warning : Invalid calibration curve, (Syringic acid)
Warning : Invalid calibration curve, (Ferulic acid)

*** End of Report ***
